# Multidrug resistance in group B Streptococcus causing urinary tract infection exposes an erythromycin-driven protective effect against oxidative stress

**DOI:** 10.1099/jmm.0.001975

**Published:** 2025-03-19

**Authors:** Devika Desai, Kelvin G. K. Goh, Sandon Ranadeera, Ellen Copeman, Matthew J. Sullivan, Glen C. Ulett

**Affiliations:** 1 School of Pharmacy and Medical Sciences, Griffith University, Gold Coast Campus, Southport 4222, QLD, Australia; 2 Institute for Biomedicine and Glycomics, Griffith University, Gold Coast Campus, Southport 4222, QLD, Australia; 3 School of Biological Sciences, University of East Anglia, Norwich NR4 7TJ, UK

**Keywords:** antibiotic resistance, group B *Streptococcus*, *Streptococcus agalactiae*

## Abstract

Multidrug resistance has been reported in group B *Streptococcus* (GBS) from various origins, but rates among urinary tract infection (UTI) isolates are largely unknown. Erythromycin, a second-line antibiotic for GBS for which high rates of resistance have been reported, was recently shown to support the resistance of *Staphylococcu*s to oxidative stress. To survey multidrug-resistant (MDR) GBS from UTI and to investigate the effect of erythromycin exposure on the bacteria’s ability to resist oxidative stress, we determined the antibacterial activity of 18 antibiotics against 292 GBS UTI isolates by disc diffusion and used i*n vitro* growth assays of MDR GBS exposed to erythromycin to examine relative resistance to oxidative stress in the form of H_2_O_2_. A high proportion of all 292 GBS isolates (33.6%) were MDR, reflecting high rates of resistance to four antibiotics: azithromycin (44.5%), clindamycin (26%), erythromycin (36.3%) and tetracycline (81.5%); however, no resistance was detected for any other antibiotics tested. Rates of resistance were not significantly different when analysed according to clinical origins (acute and recurrent UTI, asymptomatic bacteriuria). The growth of MDR GBS was attenuated and severely inhibited by exposure to erythromycin and H_2_O_2_, respectively. Surprisingly, exposure of MDR GBS to erythromycin significantly relieved the severe growth inhibitory effect of H_2_O_2_, signifying a partial rescue effect of the antibiotic. The GBS isolates in this study exhibit high levels of multidrug resistance without an association between resistance and clinical origin. Exposure of MDR GBS to erythromycin can partially counteract the severe growth inhibitory effect from H_2_O_2_.

## Introduction

Group B *Streptococcus* (GBS) is a Gram-positive commensal bacterium that resides in 30–40% of adults within the gastrointestinal and/or urogenital tract [1]. GBS causes various diseases in neonates, pregnant women, non-pregnant adults and the elderly. Among the diseases caused by GBS are urinary tract infections (UTIs), including cystitis and pyelonephritis [1]. GBS also causes asymptomatic bacteria (ABU), which is considered a risk factor in pregnant women for late gestational maternal colonization and early-onset neonatal disease [2]. GBS UTI can be refractory to antimicrobial therapy, notably in individuals with co-morbidities [3].

During infection, GBS is exposed to a variety of stressors from the host immune system, such as reactive oxygen species from phagocytes [1]. Infection is typically treated with antibiotic therapy (most often penicillin G); intrapartum antibiotic prophylaxis in pregnant women is used to prevent early-onset neonatal disease [4]. GBS isolates are normally susceptible to penicillin and other beta-lactams (e.g. ampicillin), as well as cephalosporins and carbapenems [5,7]. Alternative antibiotics for individuals who are allergic to beta-lactams are clindamycin, erythromycin, fluoroquinolones and vancomycin [8]. However, increasing rates of resistance to clindamycin, erythromycin and fluoroquinolones in GBS have been reported worldwide [4,9]. The increasing rates of resistance to macrolides and other antibiotics in *Streptococcus* spp. are, in part, related to mobile genetic elements, including integrative and conjugative elements (ICEs) that transmit antibiotic resistance [10].

The ubiquity and diversity of ICE among *Streptococcus* spp. underlie frequent genetic exchange that confers antimicrobial resistance; in GBS, for example, a mosaic ICE, *ICESag236* carrying *mef*(I) and *catQ* confers resistance to macrolides and chloramphenicol, respectively [11]. Tetracycline resistance (TET^R^) among pathogenic clones of GBS is caused by the acquisition of ICEs of the Tn916 family carrying the *tet(M*) gene [12]. Such observations support a need for continued surveillance to inform practice for antibiotic usage and study other effects of antibiotic exposure on GBS cell biology. For example, in a study of *Staphylococcus*, erythromycin increases bacterial resistance to oxidative killing [13].

Here, we examined antibiotic resistance in 292 GBS isolates collected from urine of patients with UTI and asymptomatic pregnant women. Identification of high levels of multidrug resistant (MDR) led us to explore whether exposure to erythromycin might increase the capacity of GBS to resist oxidative stress, as would be part of the host immune response to GBS during infection.

## Methods

### Bacterial isolates

GBS isolates used in this study are described previously [3]. Briefly, the isolates were cultured from urine of adults who were assessed for UTI or as part of routine screening (collected originally at University of Alabama Birmingham Hospital between August 2007 and 2012; approval X070722011, Committee on Human Experimentation; MSC/02/11/HREC, Griffith University Human Ethics Committee). For this study, 292 isolates were grouped by clinical origin of acute infection (*n*=61); recurrent (*n*=47; repeat isolates) and asymptomatic bacteriuria (*n*=184) as described in [3]. GBS were grown on 5% horse blood agar at 37 °C overnight.

### Antibiotic susceptibility testing

Antibiotic susceptibility was determined using the Kirby–Bauer disc diffusion method, according to the Clinical and Laboratory Standard Institute (CLSI) M100 (29th ed.) guidelines. *Streptococcus pneumoniae* ATCC 49619 was used for quality control. The zone of inhibition was compared to CLSI reference values to classify isolates as susceptible, intermediate or resistant. The following antibiotics with the amount of μg per disc (Oxoid) shown in parentheses were used: ampicillin (10), azithromycin (15), cefamandole (30), cefepime (30), cefotaxime (30), ceftriaxone (30), cephazolin (30), chloramphenicol (30), ciprofloxacin (5), clindamycin (2), erythromycin (15), gentamicin (120), levofloxacin (5), linezolid (30), ofloxacin (5), penicillin (10 units), tetracycline (30), vancomycin (30).

### Co-exposure of GBS to erythromycin and oxidative stress

In detecting high rates of MDR and resistance to erythromycin, we examined whether exposure of MDR GBS to erythromycin might increase the ability of the bacteria to resist oxidative stress, as recently described for *Staphylococcus aureus* [13]. The growth of GBS exposed to oxidative stress was tested using MDR strain 807 [resistant to azithromycin (AZM^R^), clindamycin (DA^R^), erythromycin (ERY^R^) and tetracycline (TET^R^); selected due to its MDR phenotype and use in multiple prior studies] [14,16]. These assays were performed in 200 µl volumes of Todd-Hewitt broth (THB) supplemented with 0.0625 µg ml erythromycin and/or 0.5 mM hydrogen peroxide (H_2_O_2_) as a source of oxidative stress. The device used to incubate GBS in the presence of ERY and H_2_O_2_ was a ClarioSTAR plate reader (BMG Labtech); the 96-well plates (Cellstar, Cat. No. 655 180, F-bottom) containing GBS in 200 µl cultures were incubated at 37 °C with agitation (300 r.p.m.), and absorbance (OD600 nm) was measured every 15 min. Control conditions included THB with GBS without antibiotic or H_2_O_2_ and THB without GBS (baseline). The assays were performed in triplicate with four independent experiments. Data shown represent means±sems of all independent experiments. The rates of resistance to each antibiotic were compared across the groups of isolates (i.e. acute UTI, recurrent UTI and asymptomatic bacteriuria) using Chi-square analysis. Statistical analyses were carried out using SPSS v26.0 and GraphPad Prism v8.0, with significance accepted at *P*<0.05.

## Results

The antibiotic resistance profiles of the 292 GBS isolates are shown in Table 1. All the isolates were uniformly susceptible to all the antibiotics tested, except azithromycin (AZM), clindamycin (DA), erythromycin (ERY) and tetracycline (TET); a high proportion of the isolates (all isolates; Table 1) were resistant to AZM (130/292, 44.5%), DA (76/292, 26%), ERY (106/292, 36.3%) and TET (238/292, 81.5%). Multidrug resistance was also common, with the rate of MDR GBS (non-susceptible to at least 1 agent from≥3 antimicrobial categories [17,18]) being 33.56% (98/292); 19.85% of the isolates were resistant to four or more antimicrobials. Unexpectedly, the proportions of isolates resistant to each antibiotic were not significantly different when analysed according to clinical origin (acute UTI, recurrent UTI, asymptomatic bacteriuria; Table 1). For example, the rates of resistance to AZM were between 42.6 and 45.7% among the clinical origin groups vs. overall rate of resistance of 44.5%; similar trends for DA (19.1–27.7% for the groups vs. 26% overall), ERY (32.8–38.6% for the groups vs. 36.6% overall) and TET (81–83% for the groups vs. 81.5% overall) showing no differences between the groups in terms of rates of antibiotic resistance. Collectively, these findings show (i) high rates of resistance to AZM, DA, ERY and TET among these GBS isolates and (ii) similar rates of resistance among the isolates regardless of clinical origin.

**Table 1.** Rates of antibiotic resistance among GBS causing acute UTI, recurrent UTI and ABU shown as percentage [number of isolates (*n*)] Isolates were classified as resistant, intermediate (Interm.) or susceptible (Suscept.) based on the disc diffusion method and CLSI guidelines. The rates of resistance to each antibiotic were compared across the groups of isolates (i.e. acute UTI, recurrent UTI and asymptomatic bacteriuria) using Chi-square analysis.

	All isolates(*n*=292)			Acute UTI isolates(*n*=61)			Recurrent UTI isolates(*n*=47)			Asymptomatic bacteriuria(*n*=184)		
	Resistant	Interm.	Suscept.	Resistant	Interm.	Suscept.	Resistant	Interm.	Suscept.	Resistant	Interm.	Suscept.
**Azithromycin**	**44.5%**(*n*=130)	2.1%(*n*=6)	53.4%(*n*=156)	42.6%*(*n*=26)	0%(*n*=0)	57.4%(*n*=35)	**42.6%**†(*n*=20)	0%(*n*=0)	57.4%(*n*=27)	**45.7%**(*n*=84)	3.2%(*n*=6)	51.1%(*n*=94)
**Clindamycin**	**26%**(*n*=76)	0%(*n*=0)	74%(*n*=216)	26.2%*(*n*=16)	0%(*n*=0)	73.8%(*n*=45)	**19.1%**†(*n*=9)	0%(*n*=0)	80.9%(*n*=38)	**27.7%**(*n*=51)	0%(*n*=0)	72.3%(*n*=133)
**Erythromycin**	**36.6%**(*n*=107)	10.3%(*n*=30)	53.1%(*n*=155)	32.8%*(*n*=20)	11.5%(*n*=7)	55.7%(*n*=34)	**34.1%**†(*n*=16)	8.5%(*n*=4)	57.4%(*n*=27)	**38.6%**(*n*=71)	10.3%(*n*=19)	51.1%(*n*=94)
**Tetracycline**	**81.5%**(*n*=238)	1.7%(*n*=5)	16.8%(*n*=49)	82%*(*n*=50)	1.6%(*n*=1)	16.4%(*n*=10)	**83%**† (*n*=39)	0% (*n*=0)	17%(*n*=8)	**81%**(*n*=149)	2.2%(*n*=4)	16.8%(*n*=31)

* Non-significant, comparing the proportion of resistance in acute UTI isolates vs. resistance in recurrent UTI isolates or asymptomatic bacteriuria isolates.

† Non-significant, comparing the proportion of resistance in recurrent UTI isolates vs. resistance in asymptomatic bacteriuria isolates.

**Fig. 1.**
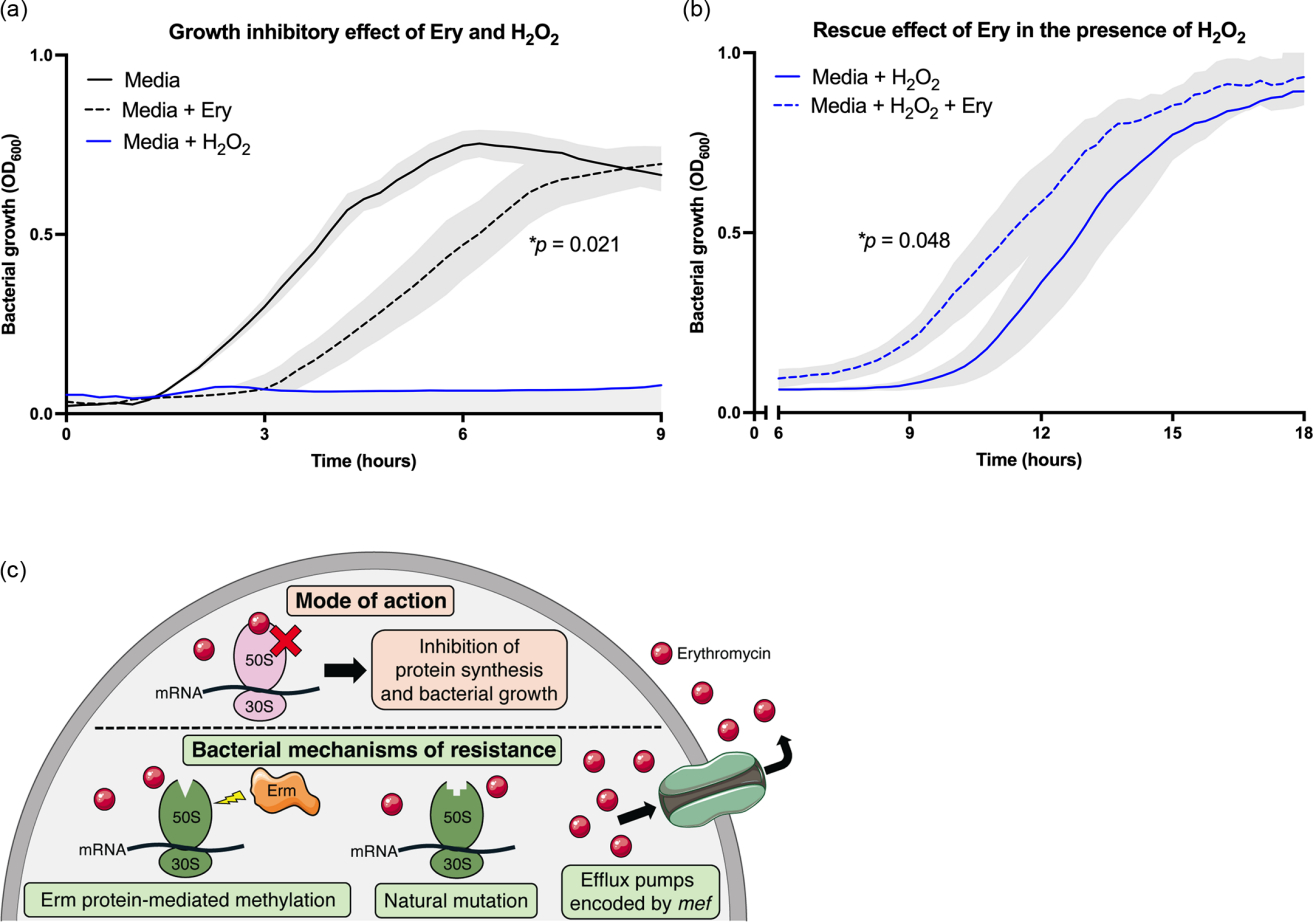
Effect of erythromycin (ERY) on H_2_O_2_-driven attenuation of growth. GBS strain 807 was grown in THB medium (black line) and compared to THB+ERY (black dashed) and THB+H_2_O_2_ (blue line) (**a**). Beyond 9 h, growth of GBS 807 in media with H_2_O_2_ (blue line) was compared to growth in media with both H_2_O_2_ and ERY (**b**). The concentrations of ERY and H_2_O_2_ used were 0.0625 µg ml^−1^ and 0.5 mM, respectively. Lines and shading show mean and sem for four independent assays; growth curves were compared using area-under-the-curve analysis followed by student’s t-test to compare test conditions to control conditions (e.g. for effect of ERY on growth of MDR GBS exposed to H_2_O_2_). Schematic illustrating the mode of action of ERY (above dotted line) vs. resistance mechanisms in bacteria (below dotted line) (c). Erythromycin binds to the 23S rRNA molecule in the 50S subunit of the bacterial ribosome, inhibiting protein synthesis. *Streptococci* resist the bacteriostatic effect of ERY using three mechanisms – (**i**) ERY methylates a conserved residue within the 50S ribosomal subunit essential for ERY binding, thus blocking ERY from binding to its target; (ii) natural mutations in genes encoding for the 23S rRNA or in ribosomal proteins L4 and L22 diminish the binding efficacy of ERY to its target; (iii) specialized efflux pumps encoded by the *mef* gene transport ERY out of the bacterial cell (as reviewed in [35]). GBS 807 harbours Tn6002, an *erm(B)*-carrying Tn916-related streptococcal element that has a ∼2.8 kb *erm(B)*-containing DNA fragment between *orf20* and *orf19* of Tn916 [27], indicating the mechanism of resistance in GBS 807 relates to ribosomal target site modification.

We next examined whether exposure to ERY might affect the ability of MDR GBS to resist oxidative stress in the form of H_2_O_2_. Growth of MDR GBS strain 807 in media supplemented with ERY was significantly attenuated vs. media alone (*P*=0.021, Fig. 1a), which compared to a complete inhibition of growth in the presence of H_2_O_2_ alone (Fig. 1a). Further analysis over an extended time course revealed a partial rescue effect of the antibiotic against H_2_O_2_-driven growth inhibition, whereby ERY relieved the inhibitory effect of H_2_O_2_ (*P*=0.048, Fig. 1b). Testing of additional ERY^R^ strains revealed similar shifts in the growth curve whereby ERY significantly relieved the growth inhibitory effect of H_2_O_2_ towards the growth of MDR GBS strain 267 (AZM^R^; DA^R^; ERY^R^; TET^R^) and GBS strain 760 (AZM^R^; DA^R^; ERY^R^; TET^R^) (Fig. S1, available in the online Supplementary Material). Taken together, these data show that exposure of MDR GBS to ERY can partially counteract the severe growth inhibitory effect of H_2_O_2_.

## Discussion

GBS is almost uniformly susceptible to penicillin and other beta-lactams [6,7], which are frequently prescribed for the treatment of UTIs [19], but rising rates of resistance to other antibiotics [4,9], including macrolides [20], highlight a need for continued surveillance of antibiotic resistance in GBS. To address this, we examined the rates of resistance in a collection of 292 GBS isolates from UTI. The key findings of the current study are as follows: (i) these isolates exhibit high levels of multidrug resistance that reflect resistance to AZM, DA, ERY and TET; (ii) the proportion of these isolates that are resistant to individual antibiotics is similar irrespective of clinical origin (i.e. from acute or recurrent UTI or asymptomatic bacteriuria); (iii) ERY partially counteracts the growth inhibitory effect of H_2_O_2_ towards MDR GBS.

The rates of AZM^R^ (44.5%), DA^R^ (26%), ERY^R^ (36.3%) and TET^R^ (81.5%) detected among the 292 GBS isolates in this study are comparable to a few prior studies. For example, in a study of 200 isolates cultured from vaginal/rectal specimens, over half of all isolates were ERY^R^ (54%) [9] and a third were resistant to DA^R^ (33%) [21]. Investigation of GBS isolates collected from pregnant women by Burcham *et al*. [22] showed that among 39 isolates, 15% were resistant to penicillin, 30.8% were DA^R^, 43.6% were ERY^R^ and 94.9% were TET^R^ [22]. Interestingly, all the penicillin-resistant isolates were of capsular serotype II and V, leading to a suggestion that penicillin resistance might be localized to particular serotypes [22]. Assefa *et al*. [23] tested 41 GBS isolates and reported high rates of resistance to penicillin (19.5%), vancomycin (17%), ampicillin (14.6%) and an MDR rate of 43.9% [23]; some such results are uncertain; however, given that for penicillin, non-susceptibility in GBS is very difficult to correctly identify with genomic testing being considered vital given known challenges with susceptibility testing [24], and vancomycin resistance in GBS is very rare, having been confirmed on only a few occasions [25]. Nonetheless, trends of increased rates for antimicrobial resistance in GBS as reported in several countries in recent years are concerning because MDR limits choice of treatment for GBS infections. In the current study, similar proportions of GBS isolates were found to be antibiotic resistant across different clinical origins (acute and recurrent UTI, asymptomatic bacteriuria). This was surprising since isolates recovered from recurrent infections can be more antibiotic resistant than isolates from non-recurrent episodes of disease. In the future, we will assess the presence of ICE-carrying determinants for antibiotic resistance among the strains examined in this study, including *erm*(TR)-carrying genetic elements [26].

Erythromycin is a second-line antibiotic that is often prescribed to those with an allergy to beta-lactams [8], and a recent study of *S. aureus* showed that exposure to ERY increased the bacteria’s resistance to oxidative stress [13], which GBS likely encounters during infection. In this context, we examined whether exposure of MDR GBS to ERY might increase GBS resistance to H_2_O_2_. Our findings show that ERY partially rescues GBS from oxidative stress when the bacteria are grown in the presence of H_2_O_2_ and the antibiotic. The mechanism underlying this effect is unclear, although the mode of action of ERY vs. resistance mechanisms is shown in Fig. 1(c). This shows the presence of Tn6002, an *erm(B)*-carrying Tn916-related streptococcal element [27] in MDR GBS 807 [15], indicating that resistance in this strain relates to ribosomal target site modification. Interestingly, the growth of GBS 807 in media supplemented with a low amount of ERY (0.0625 µg ml) was attenuated vs. media alone. Sub-inhibitory concentrations of ERY can affect various aspects of bacterial cell physiology, such as, for example, by inhibiting toxin expression in *Staphylococcus* [28]; ERY-resistant bacteria can exhibit low-fitness-cost mutations [29]. It is unclear how ERY inhibits the growth of MDR GBS strain 807.

Antibacterial agents can exert antioxidant effects in some bacteria separate from on-target effects, which can influence transcriptional and stress responses in the microbe [30]. Erythromycin was recently shown to induce antioxidant systems and glutathione in the eukaryotic microbe *Chlorella vulgaris* [31]. Interestingly, *Enterococcus* upregulates expression of the transcriptional regulator CodY when exposed to ERY [32], indicating effects on transcriptional activity in another gram-positive pathogen. Furthermore, a homologue of the CodY regulator in *S. pneumoniae* was essential to activate a global transcriptomic response to support resistance to H_2_O_2_ stress [33]. On the other hand, several antibiotics can trigger physiologically relevant generation of H_2_O_2_ in bacterial cells [34]; this has not yet been reported for ERY nor Streptococci. Another consideration is possible clinical scenarios where H_2_O_2_ and ERY might interact in an infected host undergoing antibiotic treatment; one scenario could be in subcellular niches, for example, where bacteria have been phagocytosed and the antibiotic has reached the intracellular compartment, or in areas of inflammation that could result in potentially exposing GBS to both factors (e.g. via lysis/degradation of host cells around bacteria at the site of infection where antibiotics might be co-located). In conclusion, given the role of reactive oxygen species in host antimicrobial responses, the ability of GBS to respond to survive in conditions of stress [1] and our findings that ERY can partially relieve the inhibitory effect of H_2_O_2_ on GBS growth, further work to examine the effects of antibiotics on stress responses in GBS is warranted.

## supplementary material

10.1099/jmm.0.001975 Uncited Fig. S1.

## Abbreviations

CLSI: Clinical and Laboratory Standard Institute
GBS: group B *Streptococcus*
ICEs: integrative and conjugative elements
MDR: multidrug resistant
THB: Todd-Hewitt broth
UTI: urinary tract infection
