# Supplementary material for: Multidrug resistance in group B Streptococcus causing urinary tract infection exposes an erythromycin-driven protective effect against oxidative stress
Source: J Med Microbiol. 2025 Mar 19;74(3):001975. doi: 10.1099/jmm.0.001975 (PMC11923109; doi:10.1099/jmm.0.001975)
Supplement: Uncited Fig. S1. [file jmm-74-01975-s001.pdf]

Multidrug resistance in group B streptococcus causing urinary tract infection exposes an erythromycin-driven protective effect against oxidative stress

Devika Desai[1*], Kelvin G. K. Goh[1*], Sandon Ranadeera[1], Ellen Copeman[1,2], Matthew J. Sullivan[1,2], and Glen C. Ulett[1†]

[1]School of Pharmacy and Medical Sciences, and Institute for Biomedicine and Glycomics, Griffith University, Gold Coast Campus, QLD, Australia 4222

[2]School of Biological Sciences, University of East Anglia, Norwich, NR4 7TJ, United Kingdom

Supplemental Material.

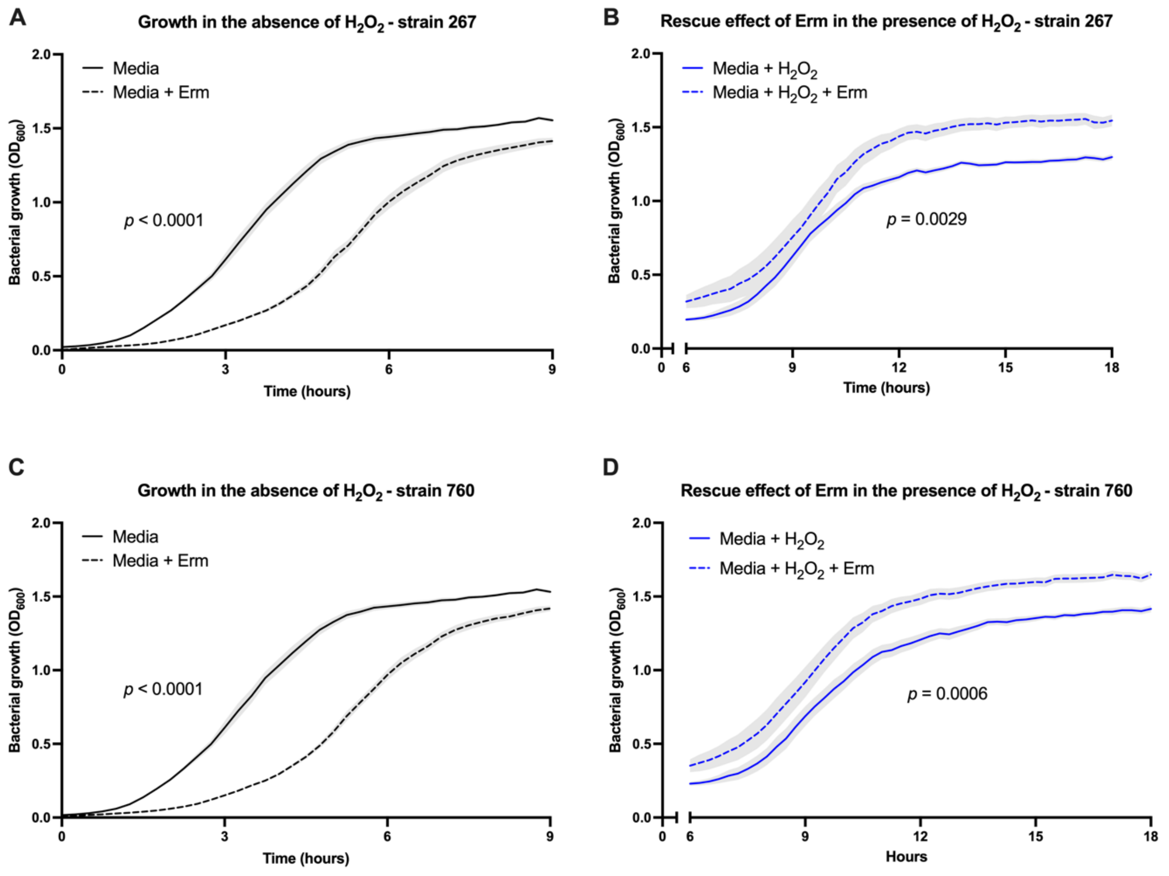

Supplementary Figure 1: Effect of erythromycin (ERY) on H_2O_2-driven attenuation of growth of GBS strain 267 and GBS strain 760. The bacteria were grown in THB medium (black line) and compared to THB + ERY (black dashed), and THB + H_2O_2 (blue line) (A). Beyond 9h, bacterial growth in media with H_2O_2 (blue line) was compared to growth in media with both H_2O_2 and ERY (B). The concentrations of ERY and H_2O_2 used were 0.0625 µg/mL and 0.5 mM, respectively. Lines and shading show mean and SEM for twelve independent assays; growth curves were compared using area-under-the-curve analysis followed by student's *t* tests to compare test conditions to control conditions (e.g., for effect of ERY on growth of MDR GBS exposed to H_2O_2. There was no significant effect of ERY on H_2O_2-driven attenuation of growth of several other GBS strains (data not shown).
